# Triacylglycerols and Fatty Acid Compositions of Cucumber, Tomato, Pumpkin, and Carrot Seed Oils by Ultra-Performance Convergence Chromatography Combined with Quadrupole Time-of-Flight Mass Spectrometry

**DOI:** 10.3390/foods9080970

**Published:** 2020-07-22

**Authors:** Yanfang Li, Fanghao Yuan, Yanbei Wu, Yaqiong Zhang, Boyan Gao, Liangli Yu

**Affiliations:** 1 Institute of Food and Nutraceutical Science, School of Agriculture and Biology, Shanghai Jiao Tong University, Shanghai 200240, China; zoe_li@sjtu.edu.cn (Y.L.); yuanfanghao@sjtu.edu.cn (F.Y.); yqzhang2006@sjtu.edu.cn (Y.Z.); 2 Beijing Advanced Innovation Center for Food Nutrition and Human Health, Beijing Technology & Business University (BTBU), Beijing 100048, China; yanbeiwu@btbu.edu.cn; 3 Department of Nutrition and Food Science, University of Maryland, College Park, MD 20742, USA; lyu5@umd.edu

**Keywords:** triacylglycerol compositions, vegetable seed oils, ultra-performance convergence chromatography (UPC^2^), quadrupole time-of-flight mass spectrometry (Q-TOF MS), fatty acid composition

## Abstract

The triacylglycerol (TAG) compositions of cucumber, tomato, pumpkin, and carrot seed oils were analyzed using ultra-performance convergence chromatography (UPC^2^) combined with quadrupole time-of-flight mass spectrometry (Q-TOF MS). A total of 36, 42, 39, and 27 different TAGs were characterized based on their Q-TOF MS accurate molecular weight and MS^2^ fragment ion profiles in the cucumber, tomato, pumpkin, and carrot seed oils, respectively. Generally, different vegetable seed oils had different TAGs compositions. Among the identified fatty acids, linoleic acid was the most abundant fatty acid in cucumber, tomato, and pumpkin seed oils and the second most abundant in carrot seed oil with relative concentrations of 54.48, 48.69, 45.10, and 15.92 g/100 g total fatty acids, respectively. Oleic acid has the highest concentration in carrot seed oil and the second highest in cucumber, tomato, and pumpkin seed oils, with relative concentrations of 78.97, 18.57, 27.16, and 33.39 g/100 g total fatty acids, respectively. The chemical compositions of TAGs and fatty acids could promote understanding about the chemical profiles of certain vegetable seed oils, thus improving the potential ability to select appropriate oils with specific functions and a high nutritional value and then develop functional foods in the future.

## 1. Introduction

Cucumber (*Cucumis sativus*), tomato (*Solanum lycopersicum*), pumpkin (*Curcubita pepo*), and carrot (*Daucus carota*) are widely consumed vegetables around the world. After industrial manufacturing, these vegetables could be processed into canned food, juice, smoothies, sauce, etc., thus they became more acceptable due to their convenience, health, nutrition, and safety [1,2]. Following industrial processing, significant amounts of vegetable seeds end up as waste, even though they have been found to contain significant amounts of healthy components such as lipids, protein, dietary fiber, and other bioactive compounds [3,4,5,6,7]. Among the nutrients in these seeds, lipid is one of the most important components, which contains more than 95% triacylglycerols (TAGs) [5,8]. Recently, increasing evidence has demonstrated that not only the fatty acid compositions, but also their binding positions in TAGs play very important roles in the functionality and nutrition values of seed oils [9,10,11,12]. For example, binding positions of fatty acids to TAG altered their oxidative stability, calcium absorption, and lipid metabolism [11]. Previous researches have investigated the fatty acid compositions of these seed oils, however very little research has been conducted on the exact chemical structures and compositions of TAGs in these seed oils, especially on the binding positions of fatty acids in TAGs [5,13,14,15,16,17,18]. Thus, in order to clarify the potential availability of developing nutritional components from these wasted seeds, it is worthwhile investigating the TAGs positional profiles and compositions of cucumber, tomato, pumpkin, and carrot seed oils.

Recently, the development of supercritical CO_2_ ultra-performance convergence chromatography (UPC^2^) provided an advanced and efficient method for the separation of low polarity compounds and was conducive to reduce the usage of organic solvents [19,20]. Besides, the high-resolution quadrupole time-of-flight mass spectrometry (Q-TOF MS) system was able to provide accurate molecular mass weight and fragment ion information of TAGs and therefore played an important role in identifying the binding position of fatty acid side chains. The combination of UPC^2^ and Q-TOF MS technology has been used to examine TAGs compositions of cow milk fat [20], sunflower, corn, soybean oils [21], and olive oils [22] due to the excellent resolution and separation ability. 

In this study, TAG composition and their binding positions, as well as the composition of free fatty acids in cucumber, tomato, pumpkin, and carrot seed oils were evaluated using UPC^2^-Q-TOF-MS and GC-MS, respectively. The relative concentrations of the identified TAGs and free fatty acids were also semi-quantified based on the area normalization method. Results from this study could improve the utilization of these seed oils as functional ingredients, along with increased profits of vegetable manufacturers and reduced environmental hazards.

## 2. Materials and Methods 

### 2.1. Materials and Regents

Cucumber, tomato, pumpkin, and carrot seed oil samples were gifted from the Botanic Innovations (Spooner, WI, USA) and stored at −20 °C before analyses. Acetonitrile, methanol, isopropanol, and ammonium formate were of LC-MS grade and purchased from Sigma-Aldrich (St. Louis, MO, USA). Fatty acid methyl ester (FAME) mix standard (contented 37 FAMEs) was purchased from NU-CHEK Inc. (Elysian, MN). Food-grade CO_2_ (purity > 99.99%) was supplied by Zhenxin Gas Co. Ltd. (Shanghai, China). Ultrapure water was purified by a Millipore Milli-Q 10 ultrapure water system (Billerica, MS, USA) with TOC value below 5 ppb and resistivity of 18.2 mΩ (25 °C). All the other chemical reagents of analytical grade were purchased from Sigma-Aldrich (St. Louis, MA, USA) and utilized without further purification.

### 2.2. Sample Preparations

The cucumber, tomato, pumpkin, and carrot seed oil samples were prepared based on previous lab protocol [21]. Briefly, 10 μL of each seed oil sample and 990 μL of acetonitrile/methanol/isopropanol (10:9:1, *v/v/v*) were vortex-mixed for 20 s and centrifuged at 2000 rpm for 5 min at an ambient temperature. After the supernatant was removed, the residue was vortex-dissolved thoroughly with 990 μL of isopropanol. The final mixture of the residue and isopropanol was used for UPC^2^-Q-TOF MS analysis and each sample was prepared in triplicates.

### 2.3. UPC^2^ System Condition

The conditions of UPC^2^ were set according to our previous manuscript [22]. A Waters Acquity ultra-performance convergence chromatography (UPC^2^) system (Milford, MA, USA) equipped with a binary pump, an oven, an auto-sampler, a back-pressure regulator, and an Acquity UPC^2^ BEH HSS C_18_ column (150 mm × 3.0 mm i.d.; 1.7 μm) was utilized for the separation of TAGs. The back pressure was 1800 psi and column oven temperature was 30 °C. The mobile phase A was pure supercritical fluid CO_2_ and the mobile phase B was methanol. The flow rate was 1.6 mL/min and the injection volume was 2.0 μL. The elution gradient started at 99% A, decreased linearly to 98.2% A over 5 min, decreased linearly to 98% A over the next 7 min, decreased linearly to 97% over the next 3 min, decreased linearly to 70% over the next minute, was held at 70% A for 3 min, and back to 99% A to re-equilibrate the column. Methanol with 0.1% ammonium formate as a compensated solvent was pumped at a flow rate of 0.3 mL/min by a Waters 1525 pump.

### 2.4. Quadrupole Time-of-Flight (Q-TOF) MS Conditions

A Waters Xevo-G2 Q-TOF MS system was utilized to characterize the chemical structures of TAGs in cucumber, tomato, pumpkin, and carrot seed oils. The electrospray ionization (ESI) in positive mode was used in a mass range from 100 to 1200 Da at a capillary voltage of 3.0 kV and a cone voltage of 50.0 V. The source offset was 80.0 V. Leucine enkephalin was used as the lock mass (m/z 556.2771 in ESI^+^). The source temperature was 120 °C and the desolvation temperature was 500 °C. The desolvation gas (nitrogen) was set at a flow of 800.0 L/h and the collision gas (argon) was set at 150.0 L/h.

Data were collected using Masslynx 4.1 software (Milford, MA, USA) in MS^E^ mode. The MS acquisition method consisted of a low collision energy mode (MS^1^) and a high collision energy mode (MS^2^). In the MS^1^ mode, the collision energy was 6 eV and parent ions were obtained. The information of fragment ions was collected in the MS^2^ mode and the collision energy was 35 eV. The scan time was 0.2/s.

### 2.5. Free Fatty Acid Composition Analysis

Free fatty acid compositions of cucumber, tomato, pumpkin, and carrot seed oils were determined by previous lab protocol [23]. Briefly, 20 mg of each oil sample was vortex mixed thoroughly with 0.4 mL of methylbenzene and 0.4 mL of KOH-MeOH (0.5 mol/L). The mixture was sealed and heated at 60 °C for 10 min. After cooling the reaction mixture to an ambient temperature, 2 mL of boron trifluoride-MeOH (14%) was added and the mixture, which was sealed with a lid, was heated at 60 °C for 5 min. Before being vortexed, 2 mL of isooctane and 3 mL of ultrapure water were added. The supernatant was injected for GC analysis. Fatty acid compositions were determined using Agilent 7890A gas chromatograph equipped with FID detector and DB-23 silica capillary column (60 m length × 0.25 mm with a 0.25 μm film thickness) using helium as the carrier gas. The injection volume was 1 μL. The oven temperature was initially at 100 °C and increased to 184 °C by 10.5 °C/min. After holding at 184 °C for 3 min, it was increased again to 240 °C at a rate of 6 °C/min. Fatty acid methyl esters were identified by the retention time compared with those of the standard FAMEs. Relative concentrations of identified fatty acids were quantified based on the area normalization method, which is calculated using each identified individual peak area divided by the total area of all peaks identified in the extracts. Each sample was analyzed in triplicate.

### 2.6. Statistical Analysis

The relative concentrations of TAGs and fatty acids were reported as the mean ± standard deviation (SD) based on the peak area normalization method. The relative concentrations of each TAG and fatty acid were analyzed with one-way ANOVA and Tukey’s post hoc test using SPSS 18.0 (Chicago, IL, USA), and *p <* 0.05 was considered a significant difference.

## 3. Results and Discussion

### 3.1. Identification of TAGs

**Figure 1:**
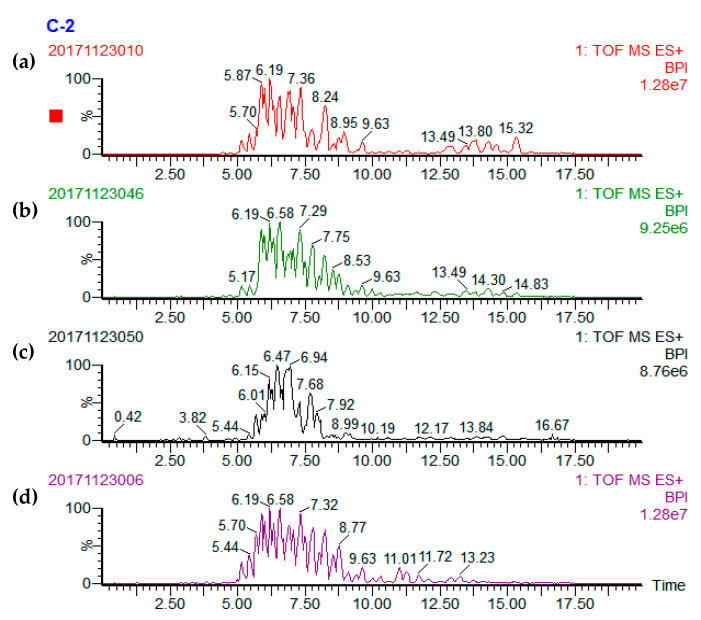
UPC^2^/Q-TOF MS BPI chromatograms of (**a**) cucumber, (**b**) pumpkin, (**c**) carrot, and (**d**) tomato seed oil samples. BPI stands for base peak integration.

**Figure 2:**
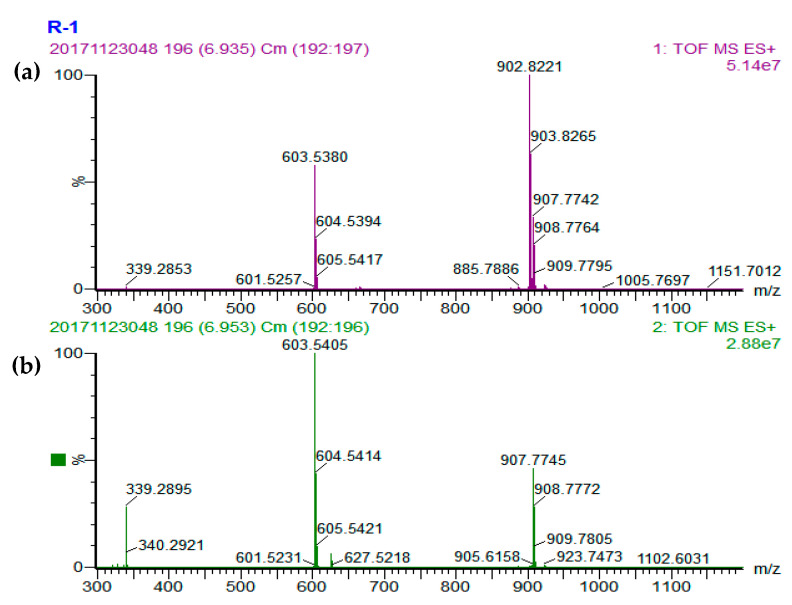
MS spectra of O-O-O. (**a**) MS^1^ spectrum and (**b**) MS^2^ spectrum.

**Figure 3:**
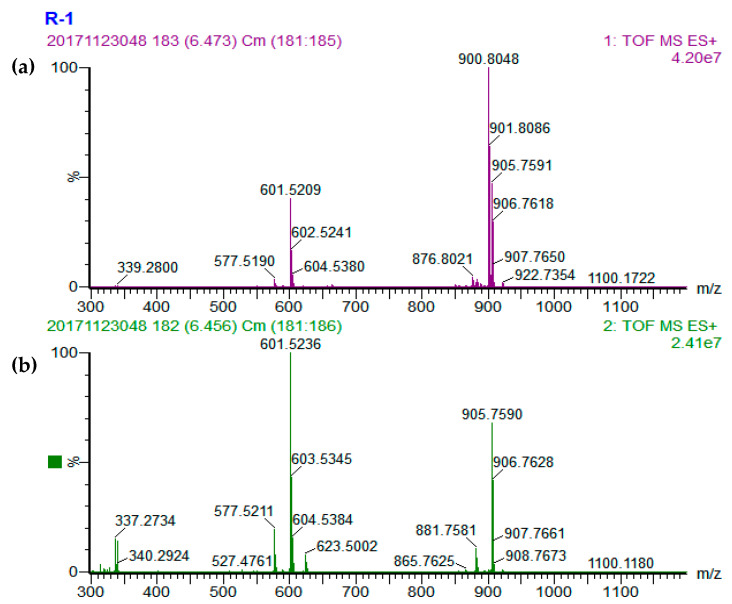
MS spectra of O-L-O. (**a**) MS^1^ spectrum and (**b**) MS^2^ spectrum.

**Figure 4:**
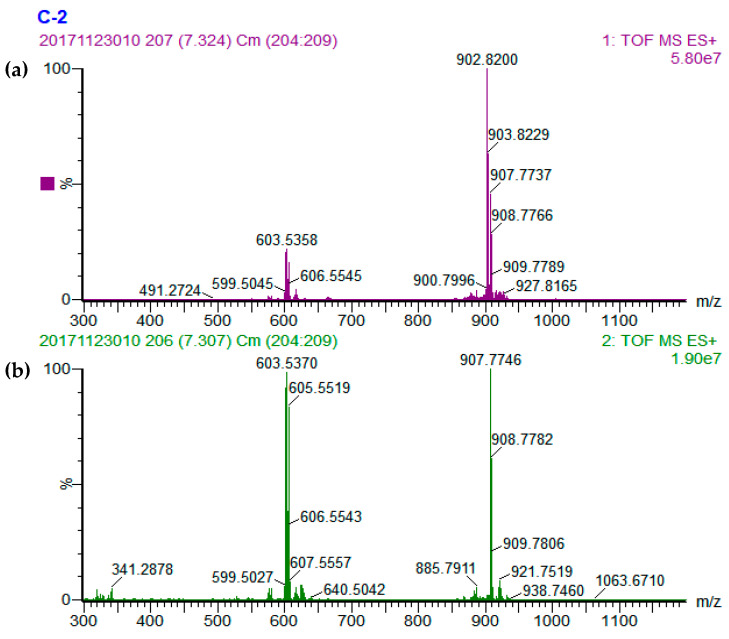
MS spectra of S-L-O. (**a**) MS^1^ spectrum and (**b**) MS^2^ spectrum.

A total of 36, 42, 39, and 27 different TAGs were tentatively identified in the cucumber, tomato, pumpkin, and carrot seed oils, respectively (Table 1). Representative base peak intensity (BPI) chromatograms of cucumber, tomato, pumpkin, and carrot seed oils obtained by UPC^2^-Q-TOF-MS are presented in Figure 1. The TAG compositions in the seed oils were determined using the accurate mass data of quasi-molecular ions of [M+NH_4_]^+^ and MS^2^ fragmentation ions of [M+H]^+^ information [21,22]. There are three typical types of TAGs: TAG that contain 3 same fatty acid side chains; or 3 different fatty acid side chains; or 2 same and 1 different fatty acid side chain with the different one at sn-2 or sn-1/sn-3 position. Three mass spectrums were presented to explain the identification of these three typical types of TAGs (Figure 2, Figure 3 and Figure 4).

Peak 25 was selected as an example to clarify the identification of TAG containing 3 same fatty acids. Peak 25 had the quasi-molecular ion of [M+NH_4_]^+^ at m/z 902.8221 (Figure 2a), referred to as the chemical formula of C_57_H_104_O_6_. The MS^2^ daughter ion of diacyl fragment in peak 25 was m/z 603.5405 ([M-RCOO+H]^+^), representing the only fragment of [O-O]^+^ (Figure 2b). The lost ion fragment had a molecular mass of 299.2773, suggesting that the lost fatty acid was oleic acid. Therefore, peak 25 was identified to be O-O-O.

Peak 23 was selected as an example to explain the identification of TAG containing 2 different fatty acids. Peak 23 had the quasi-molecular ion of [M+NH_4_]^+^ at m/z 900.8048 (Figure 3a), referred to as C_57_H_102_O_6_. The MS^2^ daughter ion of diacyl fragments in peak 23 were m/z 603.5345 and 601.5236 ([M-RCOO+H]^+^) (Figure 3b), representing the fragments of [O-O]^+^ and [O-L]^+^ with relative natural abundances of 41% and 100%, respectively. It has been previously proved that fatty acids on sn-2 position binding to TAGs have greater bond energy and are harder to be eliminated in MS^2^ collision energy mode than those on sn-1 and sn-3 positions [20,21,22,24,25]. The ratio of [O-L]^+^ and [O-O]^+^ was over 2:1, suggesting that peak 23 could be tentatively identified as O-L-O.

Peak 27 was selected as an example for clarifying the identification of a TAG containing 3 different fatty acids. Peak 27 had the quasi-molecular ion of [M+NH_4_]^+^ at m/z 902.8200 (Figure 4a), referred to as C_57_H_104_O_6_. The MS^2^ daughter ion of diacyl fragments in peak 27 were m/z 601.5211, 603.5370, and 605.5519 ([M-RCOO+H]^+^) (Figure 4b), representing the fragments of [O-L]^+^, [L-S]^+^, and [O-S]^+^, with relative natural abundances of 90.25%, 96.33%, and 81.91%, respectively. In consideration of the MS rules regarding the fatty acid position and their bond energies mentioned above, linoleic acid was assigned to sn-2 position since the relative abundance of [O-S]^+^ was the lowest in nature. Then, peak 27 was identified as S-L-O. 

A total of 36 TAGs were tentatively identified in cucumber seed oil, with SLO as the most abundant TAG, followed by LLO and LLL (Table 1). Further, 23 of the identified TAGs, namely PLPo, LnPoL, PLnLn, PoLL, PLLn, LnLnLn, SLS, SOS, LLG, LLA, OLG, LOA, OOA, SLA, OSA, LLB, OLB, LSB, LLT, OLT, LLLi, LOLi, and LLH, were reported in cucumber seed oil for the first time. By extrapolating the ratios of increments for fatty acids to the HPLC retention of component TAGs, Deineka and others found OLLn, OOO, and PPO in cucumber seed oil 20- and 30-days after fruit setting, which were not detected in the present study [26]. Different TAG compositions in cucumber seed oil may be due to different identification techniques as well as different ripening and growing conditions of the cucumber samples.

A total of 42 TAGs were detected and identified in tomato seed oil, with LLL, SOL, and OLL as the three major TAGs (Table 1). Among all the identified TAGs, 33 of them including SOO, SLS, PLS, LLLn, SOS, LLA, LOA, PLP, PoLL, PLPo, MLL, LSA, LLLi, PLLn, LSB, OOA, LOLi, LLB, LLH, OSA, LnPoL, SLE, LLnLn, LLG, OLG, LnLA, PLnLn, OOB, LLT, LnLB, LnLLi, LnLH, and OLT were reported in tomato seed oil for the first time. Silva and others calculated the theoretical compositions of 5 triacylglycerols in tomato seed oil according to a computer program developed based on the fatty acid profile by Antoniosi Filho, Mendes, and Lanças [27], which were all directly detected and identified in the present study [7]. Compared with the identification strategy of TAGs based on the fatty acid compositions, UPC^2^-Q-TOF MS technology not only directly showed higher accuracy and feasibility, but also provided the actual structure of TAGs, which is not limited by the detection of fatty acid composition. 

A total of 39 TAGs were identified in pumpkin seed oil and OSL, LOO, and OLL were the three major TAGs, with a relative concentration of 9.37, 9.18, and 7.97 g/100 g TAGs, respectively (Table 1). Among all the 39 TAGs, 22 of them, namely OLA, LLA, LSA, OOA, OLB, PoOO, PoLL, LLB, MLL, PoLO, OLG, LSB, LLLi, OSA, LOLi, LLG, OOB, PLnLn, LnPoL, LLT, OLT, and LLH were reported in pumpkin seed oil for the first time. On the other hand, LnLnLn, LLnLn, OLnLn, OLLn, OOLn, SLLn, POLn, POP, POS, and PPS have been reported in previous results [26,28,29,30,31,32] but not detected in this study. These differences might due to the different varieties of the seed samples and the different identification techniques that were used.

Similarly, a total of 27 TAGs were detected in carrot seed oil, with OOO, OLO, and OLL having the highest concentrations of 22.53, 15.33, and 10.27 g/100 g TAGs, respectively (Table 1). In agreement with a previous study, Giuffrè found that OOO was the highest TAG in all detected virgin and extra virgin olive oil and constituted 30–50% of the total TAGs by detecting the TAG content of 10 different cultivars grown in South West Calabria [33]. Considering that olive oil is a vegetable oil with many recognized biological properties [34], carrot seed oil may have similar bioactivates but warrants additional research to reveal its specific health effects. There are 17 TAGs, including OSL, OOG, PoOO, OLG, OLA, PPoO, PoLL, LLLn, MOL, OOB, OEO, PLP, OLE, OLB, LLnLn, LnLB, and LOLi, that were reported in carrot seed oil for the first time. Thao and others found 10 TAGs in carrot seed oil using LC-Q-TOF MS and seven of them, including OOO, OLO, OSO, POO, LLL, PoLO, and OOA, were also detected in the present study, whereas OLLn, LAA, and LnLnLn were not observed [35]. 

### 3.2. Fatty Acid Compositions

Four major fatty acids, including palmitic acid (C16:0), stearic acid (C18:0), oleic acid (C18:1), and linoleic acid (C18:2) were identified and their relative concentrations for each seed oil sample were investigated and are listed in Table 2. Linoleic acid was the most abundant fatty acid in cucumber, tomato, and pumpkin seed oils and the second abundant in carrot seed oil, with relative concentrations of 54.48, 48.69, 45.10, and 15.92 g/100 g total fatty acids, respectively (Table 2). The results were consistent with previous studies that linoleic acid had the highest content of 68.1%, 54.7%, and 56.4% in cucumber, tomato, and pumpkin seed oils, respectively [5,26]. Conjugated linoleic acid has been associated with several vital biological functions such as antitumor, anti-obesity, antidiabetic, anti-inflammatory, and cardioprotective activities [36,37,38], suggesting that the consumption of cucumber, tomato, and pumpkin seed oils in daily life might be good for human health, but more research is needed to verify its accuracy. In addition, oleic acid has the highest concentration in carrot seed oil and was the second abundant in cucumber, tomato, and pumpkin seed oils, with relative concentrations of 78.97, 18.57, 27.16, and 33.39 g/100 g total fatty acids, respectively (Table 2). The content of oleic acid was in agreement with previous reports, contributing about 68.1–81.2% in carrot seed oil [13,35]. As we know, oleic acid is not only the most important monounsaturated fatty acid in the diet, but also the predominate constituent of plasma free fatty acids with many bioactivities such as anti-tumor, anti-inflammatory, and cardioprotective functions [34,39,40,41]. For an extra virgin olive oil, oleic acid has to be in the 55–83% range of the total fatty acids [42,43], which is widely considered as a healthy oil. With many properties such as anti-oxidant, anti-inflammatory, and cardiovascular protective effects [34]. Consumption of carrot seed oil might have similar health effects as olive oil, but more investigation is needed in the future. Palmitic acid was the third abundant fatty acid in cucumber, tomato, pumpkin, and carrot seed oils, with concentrations of 14.98, 16.81, 14.21, and 5.07 g/100 g total fatty acids, respectively (Table 2). The content of stearic acid in cucumber, tomato, pumpkin, and carrot seed oils were 11.97, 7.34, 7.29, and 1.04 g/100 g total fatty acids, respectively (Table 2). Some minor content fatty acids such as linolenic, behenic, arachidic, lignoceric, eicosenoic, palmitoleic, and myristic acids were detected in previous studies in these seed oils, however they were not detected in this study [5,13,16,35]. This might be due to the fact that the content of these fatty acids (FAs) was under a detectable level in the present study, which may be caused by different cultivars or different harvest times of these seed samples [44].

In the comparison of fatty acid compositions determined using gas chromatography directly and calculated from TAGs compositions (Table 3), 7 of 16 relative deviations (RDs) were less than 8%, which means that most of the TAGs compositions identified in this study were consistent with their fatty acid composition results. But there was still one result with an abnormal RD value of stearic acid in carrot seed oil greater than 70%, which might be due to its low relative concentration of 1.04 g/100 g total fatty acids. The relative deviations of oleic acid and linoleic acid indicated good consistency between the determined value and calculated value in cucumber (−2.01%, 2.23%), pumpkin (−0.24%, 6.22%), and tomato (3.74%, 2.94%) seed oils. These results showed that the determined value was close to the calculated value in general.

## 4. Conclusions

In summary, a total of 36, 42, 39, and 27 different TAGs were identified in the cucumber, tomato, pumpkin, and carrot seed oils, respectively. A total of 23, 33, 22, and 17 TAGs detected in cucumber, tomato, pumpkin, and carrot seed oils were reported for the first time. Generally, different vegetable seed oils contained different TAGs compositions. SLO, LLL, OSL, and OOO were the most abundant TAGs in cucumber, tomato, pumpkin, and carrot seed oil, with relative concentrations of 11.55, 7.52, 9.37, and 22.53 g/100 g TAG, respectively. Among the identified fatty acids, linoleic acid concentration was the highest in cucumber, tomato, and pumpkin seed oils and the second highest in carrot seed oil, with relative concentrations of 54.48, 48.69, 45.10, and 15.92 g/100 g total fatty acids, respectively. Oleic acid was the most abundant in carrot seed oil and the second most abundant in cucumber, tomato, and pumpkin seed oils, with relative concentrations of 78.97, 18.57, 27.16, and 33.39 g/100 g total fatty acids, respectively. UPC^2^-Q-TOF MS technology was utilized to analyze the TAGs in four vegetable seed oils and showed higher accuracy and feasibility than the identification strategy based on the fatty acid composition. These results could provide a reference for selecting appropriate oils with specific functions and high nutritional values to develop functional foods in the future. 

## Figures and Tables

**Table 1:** Identification and relative concentration of triacylglycerols in cucumber, pumpkin, carrot, and tomato seed oils.

Peak No.	Rt (min)	Observed ([M+NH_4_]^+^)	Chemical Formula	Possible Structure	UB	TAGs Composition (g/100 g TAGs)			
						Cucumber	Tomato	Pumpkin	Carrot
1	5.17	844.7394	C_53_H_94_O_6_	M-L-L	4	1.49b ± 0.12	1.56b ± 0.03	0.85a ± 0.01	nd
2	5.45	846.7551	C_53_H_96_O_6_	M-O-L	3	nd	nd	1.03b ± 0.09	0.51a ± 0.02
3	5.52	846.7550	C_53_H_96_O_6_	P-L-Po	3	0.93a ± 0.06	1.79b ± 0.04	nd	nd
4	5.76	848.7707	C_53_H_98_O_6_	P-Po-O	2	nd	nd	nd	0.60a ± 0.01
5	6.05	848.7707	C_53_H_98_O_6_	P-L-P	2	2.82d ± 0.01	2.11b ± 0.08	2.31c ± 0.06	0.34a ± 0.00
6	5.27	868.7394	C_55_H_94_O_6_	Ln-Po-L	6	0.56b ± 0.04	0.59b ± 0.05	0.17a ± 0.02	nd
7	5.59	868.7393	C_55_H_94_O_6_	P-Ln-Ln	6	0.34c ± 0.01	0.19a ± 0.00	0.22b ± 0.01	nd
8	5.44	870.7551	C_55_H_96_O_6_	Po-L-L	5	1.83c ± 0.16	2.08d ± 0.09	0.96b ± 0.11	0.59a ± 0.02
9	5.76	870.7554	C_55_H_96_O_6_	P-L-Ln	5	1.11b ± 0.02	1.50c ± 0.04	0.37a ± 0.02	nd
10	6.01	872.7707	C_55_H_98_O_6_	P-L-L	4	7.27d ± 0.17	4.95b ± 0.25	5.62c ± 0.20	2.54a ± 0.05
11	5.70	872.7715	C_55_H_98_O_6_	Po-L-O	4	nd	nd	0.67a ± 0.08	3.38b ± 0.11
12	6.33	874.7864	C_55_H_100_O_6_	P-O-L	3	5.30a ± 0.18	5.30a ± 0.17	5.48a ± 0.07	5.83b ± 0.03
13	6.01	874.7864	C_55_H_100_O_6_	Po-O-O	3	nd	nd	1.03a ± 0.17	3.79b ± 0.06
14	7.07	876.8020	C_55_H_102_O_6_	P-L-S	2	5.59b ± 0.12	4.20a ± 0.07	3.98a ± 0.10	nd
15	6.65	876.8021	C_55_H_102_O_6_	P-O-O	2	1.20a ± 0.06	3.70b ± 0.07	3.70b ± 0.10	5.23c ± 0.05
16	5.33	890.7238	C_57_H_92_O_6_	Ln-Ln-Ln	9	0.23a ± 0.02	nd	nd	nd
17	5.55	892.7394	C_57_H_94_O_6_	L-Ln-Ln	8	nd	0.55b ± 0.03	nd	0.17a ± 0.01
18	5.70	894.7551	C_57_H_96_O_6_	L-L-Ln	7	2.07b ± 0.08	3.97c ± 0.17	0.63a ± 0.06	0.52a ± 0.02
19	5.87	896.7707	C_57_H_98_O_6_	L-L-L	6	10.17d ± 0.09	7.52c ± 0.11	7.26b ± 0.10	3.38a ± 0.09
20	6.19	898.7864	C_57_H_100_O_6_	O-L-L	5	11.16d ± 0.10	7.05a ± 0.29	7.97b ± 0.25	10.27c ± 0.06
21	6.58	900.8021	C_57_H_102_O_6_	L-O-O	4	6.83b ± 0.28	5.62a ± 0.06	9.18c ± 0.34	nd
22	6.90	900.8020	C_57_H_102_O_6_	L-S-L	4	8.93c ± 0.09	4.93a ± 0.11	5.47b ± 0.11	nd
23	6.47	900.8019	C_57_H_102_O_6_	O-L-O	4	nd	nd	nd	15.33a ± 0.13
24	7.29	902.8178	C_57_H_104_O_6_	O-S-L	3	nd	nd	9.37b ± 0.18	6.14a ± 0.10
25	6.94	902.8177	C_57_H_104_O_6_	O-O-O	3	nd	2.70a ± 0.08	4.94b ± 0.19	22.53c ± 0.24
26	7.32	902.8181	C_57_H_104_O_6_	S-O-L	3	nd	7.46a ± 0.27	nd	nd
27	7.36	902.8177	C_57_H_104_O_6_	S-L-O	3	11.55a ± 0.25	nd	nd	nd
28	7.75	904.8333	C_57_H_106_O_6_	O-S-O	2	nd	nd	nd	7.75a ± 0.24
29	7.78	904.8340	C_57_H_106_O_6_	S-O-O	2	1.85a ± 0.11	6.30b ± 0.20	6.86c ± 0.11	nd
30	8.24	904.8334	C_57_H_106_O_6_	S-L-S	2	7.64c ± 0.12	5.31b ± 0.06	4.62a ± 0.09	nd
31	8.77	906.8490	C_57_H_108_O_6_	S-O-S	1	2.36a ± 0.02	3.93c ± 0.18	2.80b ± 0.12	nd
32	7.22	926.8177	C_59_H_104_O_6_	L-L-G	5	0.57b ± 0.01	0.54b ± 0.03	0.42a ± 0.01	nd
33	7.75	926.8177	C_59_H_104_O_6_	Ln-L-A	5	nd	0.23a ± 0.02	nd	nd
34	8.00	928.8333	C_59_H_106_O_6_	L-L-A	4	1.36a ± 0.03	2.73c ± 0.11	2.23b ± 0.01	nd
35	7.64	928.8334	C_59_H_106_O_6_	O-L-G	4	0.25a ± 0.00	0.45b ± 0.01	0.58c ± 0.02	2.56d ± 0.05
36	8.53	930.8490	C_59_H_108_O_6_	L-O-A	3	1.66a ± 0.11	2.64b ± 0.10	nd	nd
37	7.96	930.8481	C_59_H_108_O_6_	O-O-G	3	nd	nd	nd	4.87a ± 0.08
38	8.53	930.8499	C_59_H_108_O_6_	O-L-A	3	nd	nd	3.33b ± 0.18	0.73a ± 0.01
39	9.63	932.8646	C_59_H_110_O_6_	L-S-A	2	nd	1.54a ± 0.07	1.58a ± 0.15	nd
40	9.06	932.8649	C_59_H_110_O_6_	O-O-A	2	0.22a ± 0.01	1.06b ± 0.02	1.39c ± 0.09	1.29c ± 0.03
41	9.63	932.8651	C_59_H_110_O_6_	S-L-A	2	1.98a ± 0.11	nd	nd	nd
42	10.30	934.8803	C_59_H_112_O_6_	O-S-A	1	0.21a ± 0.01	0.74c ± 0.04	0.53b ± 0.05	nd
43	9.06	954.8490	C_61_H_108_O_6_	Ln-L-B	5	nd	0.17a ± 0.01	nd	0.16a ± 0.00
44	9.37	956.8646	C_61_H_110_O_6_	L-L-B	4	0.43a ± 0.01	0.81b ± 0.05	0.96b ± 0.12	nd
45	8.77	956.8662	C_61_H_110_O_6_	O-L-E	4	nd	nd	nd	0.26a ± 0.01
46	9.94	958.8803	C_61_H_112_O_6_	O-L-B	3	0.28a ± 0.01	nd	1.08b ± 0.09	0.22a ± 0.01
47	9.27	958.8807	C_61_H_112_O_6_	O-E-O	3	nd	nd	nd	0.40a ± 0.01
48	10.01	958.8802	C_61_H_112_O_6_	S-L-E	3	nd	0.56a ± 0.03	nd	nd
49	10.65	960.8959	C_61_H_114_O_6_	O-O-B	2	nd	0.19a ± 0.03	0.41b ± 0.04	0.47b ± 0.01
50	11.25	960.8964	C_61_H_114_O_6_	L-S-B	2	0.62a ± 0.04	1.32b ± 0.06	0.58a ± 0.05	nd
51	10.16	970.8803	C_62_H_112_O_6_	L-L-T	4	0.12a ± 0.02	0.17b ± 0.01	0.15ab ± 0.02	nd
52	10.83	972.8959	C_62_H_114_O_6_	O-L-T	3	0.05a ± 0.00	0.10b ± 0.00	0.13c ± 0.01	nd
53	10.58	982.8803	C_63_H_112_O_6_	Ln-L-Li	5	nd	0.15a ± 0.01	nd	nd
54	11.01	984.8976	C_63_H_114_O_6_	L-L-Li	4	0.53a ± 0.03	1.51b ± 0.06	0.56a ± 0.05	nd
55	11.72	986.9116	C_63_H_116_O_6_	L-O-Li	3	0.28b ± 0.02	0.93d ± 0.01	0.49c ± 0.02	0.14a ± 0.00
56	12.42	1010.9116	C_65_H_116_O_6_	Ln-L-H	5	nd	0.11a ± 0.01	nd	nd
57	12.91	1012.9272	C_65_H_118_O_6_	L-L-H	4	0.22b ± 0.01	0.75c ± 0.04	0.08a ± 0.00	nd

TAGs stands for triacylglycerols; RT represents retention time; UB represents the number of unsaturated double bonds; nd represents not detectable; P: palmitic acid, Po: palmitoleic acid, M: myristic acid, S: stearic acid, O: oleic acid, L: linoleic acid, Ln: linolenic acid, G: gondoic acid, A: arachidic acid, B: behenic acid, Li: lignoceric acid, E: Erucic acid, T: Tricosanoic acid, H: Hexacosanoic acid. X-X-Y, X-Y-X, X-X-X, and X-Y-Z represent structures of triacylglycerols, for example, S-P-O stands for the structure of 1/3-stearoyl-2-palmitoyl-1/3-oleoylglycerol. The relative concentration of each triacylglycerol is reported as grams of triacylglycerols/100 g of oil samples. Cucumber, tomato, pumpkin, and carrot seed oils were analyzed in triplicate and results reported as mean ± standard deviation (SD). Different letters represent significant differences within a column (*p <* 0.05).

**Table 2:** Identification and relative concentration of fatty acid compositions in cucumber, pumpkin, carrot, and tomato seed oil samples.

Fatty Acid	C:D	Fatty Acid Composition (g/100 g total FAs)			
		Cucumber	Tomato	Pumpkin	Carrot
Palmitic acid	16:0	14.98b ± 0.35	16.81b ± 0.43	14.21b ± 0.05	5.07a ± 0.08
Stearic acid	18:0	11.97c ± 0.70	7.34b ± 1.16	7.29b ± 0.32	1.04a ± 0.03
Oleic acid	18:1	18.57a ± 0.17	27.16c ± 0.52	33.39d ± 0.10	78.97e ± 0.29
Linoleic acid	18:2	54.48e ± 0.85	48.69d ± 0.25	45.10c ± 0.04	15.92b ± 0.18

FAs stands for fatty acids; C:D represents carbon number:double bounds number. The relative concentration of each fatty acid is reported as grams of fatty acids/100 g of total fatty acids. Cucumber, pumpkin, carrot, and tomato seed oils were analyzed in triplicate and results are reported as mean ± standard deviation (SD). Different letters represent significant differences within a column (*p <* 0.05).

**Table 3:** Comparison of relative concentration of fatty acid compositions determined by GC and calculated by triacylglycerols compositions in cucumber, pumpkin, carrot, and tomato seed oil samples.

Fatty Acid	Fatty Acid Composition (g/100 g total FAs)											
	Cucumber			Tomato			Pumpkin			Carrot		
	MV	CV	RD	MV	CV	RD	MV	CV	RD	MV	CV	RD
Palmitic acid	14.98	9.95	50.55%	16.81	10.01	67.93%	14.21	8.8	61.48%	5.07	5.33	−4.88%
Stearic acid	11.97	17.81	−32.79%	7.34	16.43	−55.33%	7.29	15.27	−52.26%	1.04	4.98	−79.12%
Oleic acid	18.57	18.95	−2.01%	27.16	26.18	3.74%	33.39	33.47	−0.24%	78.97	63.26	24.83%
Linoleic acid	54.48	53.29	2.23%	48.69	47.3	2.94%	45.1	42.46	6.22%	15.92	26.43	−39.77%

MV, mean value of fatty acid compositions determined by GC; CV, calculated value of fatty acid compositions calculated from triacylglycerols compositions reported in Table 1; RD, relative deviation.
